# The crossroads of inflammation and nutrition: predicting neoadjuvant immunochemotherapy efficacy in esophageal squamous cell carcinoma patients

**DOI:** 10.3389/fimmu.2025.1663268

**Published:** 2025-11-20

**Authors:** Guisheng Zhang, Bingxin Gong, Yusheng Guo, Jie Lou, Ying Peng, Siqian Cai, Zhichao Fu, Yuanji Xu, Lian Yang

**Affiliations:** 1 Department of Radiology, Union Hospital, Tongji Medical College, Huazhong University of Science and Technology, Wuhan, China; 2 Hubei Provincial Clinical Research Center for Precision Radiology and Interventional Medicine, Wuhan, China; 3 Hubei Key Laboratory of Molecular Imaging, Wuhan, China; 4 College of Medical Technology and Engineering, Henan University of Science and Technology, Luoyang, China; 5 Department of Radiation Oncology, Clinical Oncology School of Fujian Medical University, Fujian Cancer Hospital, Fuzhou, China; 6 Department of Radiotherapy, Fuzong Clinical Medical College (900th Hospital), Fujian Medical University, Fuzhou, Fujian, China

**Keywords:** esophageal squamous cell carcinoma, neoadjuvant, immunochemotherapy, pathological complete response, systemic immune-inflammation index, prognostic nutritional index

## Abstract

**Background:**

Neoadjuvant immunochemotherapy (nICT) improves outcomes in esophageal squamous cell carcinoma (ESCC); however, patient response heterogeneity limits its clinical benefit. The aim of this study was to investigate the predictive value of the systemic immune-inflammation index and prognostic nutritional index score (SII-PNI score), which is jointly constructed from the systemic immune-inflammation index (SII) and prognostic nutritional index (PNI), for pathological complete response (pCR) and survival outcomes.

**Methods:**

This retrospective study included patients with stage II to IV ESCC who received nICT therapy at Wuhan Union Hospital (WHUH), Fujian Cancer Hospital (FJCH), and 900th Hospital of the Joint Service Support Force of the People’s Liberation Army of China (900th Hospital). The predictive performance of SII-PNI score was validated using receiver operating characteristic (ROC) curves. Multivariate logistic regression confirmed SII-PNI score as an independent predictor of pCR. Additionally, Cox regression model and Kaplan-Meier survival curves were employed to analyze disease-free Survival (DFS) and overall survival (OS).

**Results:**

A total of 345 patients were included, stratified into 0-score (n = 70), 1-score (n = 149), and 2-score (n = 126) groups. The combined cohort (Combined from FJCH cohort, WHUH cohort, 900th Hospital cohort) area under the ROC curve (AUC) revealed that SII-PNI score (AUC = 0.803) outperformed SII (AUC = 0.679, DeLong’s test *P* < 0.001) and PNI (AUC = 0.667, DeLong’s test *P* < 0.001) in predicting pCR. Compared with 0-score patients, those with 1-score (odds ratio [OR] = 0.159, 95% confidence interval [CI]:0.080-0.319, *P* < 0.001) and 2-score (OR = 0.025, 95% CI:0.009-0.073, *P* < 0.001) had significantly lower pCR rates. The 2-score group showed had shorter of DFS (hazard ratio [HR] = 2.487, 95% CI:1.414-4.374, *P* = 0.002) and OS (HR = 4.473, 95% CI:2.138-9.357, *P* < 0.001) versus the 0-score group.

**Conclusions:**

This study demonstrated for the first time that the SII-PNI score can be used as an independent predictor of pCR in patients with ESCC treated with nICT and has prognostic stratification value. This suggests that it has the potential to be a pre-treatment assessment tool for evaluating treatment response and prognosis before nICT treatment.

## 1 Introduction

Esophageal cancer (EC) is the seventh most common malignant tumor and the sixth most deadly cancer worldwide (1, 2). It has two main histologic subtypes: esophageal squamous cell carcinoma (ESCC) and esophageal adenocarcinoma (EAC) (3). ESCC is particularly prominent in China, ranking sixth and fourth in incidence and mortality, respectively, and accounting for approximately more than 50% of the global incidence and mortality of esophageal cancer (4–6). nICT has gained increasing attention as a therapeutic strategy for locally advanced ESCC, with several clinical studies confirming its safety and efficacy in locally advanced esophageal cancer (7–9). pCR has been shown to be an independent predictor for evaluating patients’ long-term survival (10, 11). Non-pCR patients after neoadjuvant therapy may experience worse survival outcomes due to cumulative treatment-related toxicities and increased post-treatment complications (12). This highlights the potential clinical value of predicting pCR to guide individualized treatment strategies post-nICT.

Currently, clinical predictive biomarkers for pathological response following nICT remain limited. Although programmed death-ligand 1 (PD-L1) expression, tumor mutational burden (TMB), and circulating tumor DNA (ctDNA) status can predict pathological response after nICT treatment, this multi-group biomarker-based approach is costly and demonstrates limited predictive efficacy (8, 13). Presently, the relationship between immune-inflammatory status, nutritional indicators, and tumor microenvironment and the prognosis of malignant tumors has attracted increasing attention in the academic community (14–16). Existing studies have shown that body mass index (BMI), neutrophil-lymphocyte ratio (NLR), systemic inflammatory response index (SIRI), systemic immunoinflammatory index (SII), and prognostic nutritional index (PNI) are correlated with clinical benefit and survival prognosis of esophageal cancer patients (15, 17, 18). Feng et al. (19) developed a comprehensive nutritional index (CNI) by integrating BMI, Usual Body Weight Percentage (UBWP), Total Lymphocyte Count (TLC), Albumin (ALB), and Hemoglobin (HB) to predict the pCR rate in patients with ES following nICT. However, the model incorporated only nutritional indicators and not immunoinflammatory parameters, the model failed to fully reflect systemic nutritional and inflammatory status, and it had limited predictive power (CNI AUC = 0.666).

Currently, nICT as a treatment strategy for ESCC has been widely recognized for its clinical efficacy and safety. However, highly sensitive and reliable biomarkers for predicting pCR rates following nICT remain lacking. Furthermore, the impact of baseline SII-PNI scores on the therapeutic efficacy of nICT and clinical outcomes in ESCC patients has not been elucidated. Therefore, we evaluated the pCR rates in ESCC patients with different SII-PNI scores (0, 1, and 2) after nICT and further analyzed the correlation between SII-PNI scores and pCR rates, as well as prognosis.

## 2 Materials and methods

This retrospective cohort study was approved by the Ethics Committee of Tongji Medical College of Huazhong University of Science and Technology, the Ethics Committee of Fujian Cancer Hospital, and the Ethics Committee of 900th Hospital of the Joint Service Support Force of the People’s Liberation Army of Chinas. The study strictly adhered to the code of good clinical practice and the ethical guidelines of the Declaration of Helsinki, and written informed consent was waived with the approval of the institutional review boards of the participating centers based on the characteristics of retrospective studies and the principle of patient data anonymization.

### 2.1 Study design and patient selection

This was a multicenter retrospective study that included 106 patients treated at Wuhan Union Hospital (WHUH) between October 2020 and December 2023, 204 patients treated at Fujian Cancer Hospital (FJCH) between September 2019 and December 2024, and 35 patients treated at 900th Hospital of the Joint Service Support Force of the People’s Liberation Army of China (900th Hospital) between February 2020 and July 2023, with a total of 78 (22.6%) patients achieving pCR after nICT treatment. All enrolled patients had stage II to IV ESCC and received nICT followed by radical surgery. Inclusion criteria: (1) Pathologically confirmed diagnosis of stage II to IV esophageal cancer; (2) Radical surgery with available pathology after undergoing nICT; (3) Age ≥ 18 years. Exclusion criteria (1) Non-radical resection (R1/R2 resection) after undergoing nICT; (2) Other pathological types of EC (non-ESCC); (3) Concomitant or pre-existing malignant tumors at other sites; (4) incomplete prognostic data.

### 2.2 Procedures

We retrospectively collected baseline data on clinical case patients, including patient demographics (age, gender, body mass index [BMI], Eastern Cooperative Oncology Group Performance Status [ECOG PS] score, hypertension, smoking, drinking), biochemical data (carcinoembryonic antigen [CEA], total bilirubin [TBIL], alanine aminotransferase [ALT], aspartate aminotransferase [AST], alkaline phosphatase [ALP], albumin [ALB], globulin [GLOB], creatinine [CRE], urea nitrogen [BUN], fasting blood glucose [FBG], total cholesterol [TC], triglycerides [TG], high-density lipoprotein cholesterol [HDL-C], low-density lipoprotein cholesterol [LDL-C], lactate dehydrogenase [LDH], calcium [Ca], white blood cell count [WBC], hemoglobin [HGB], platelet count [PLT], neutrophil count [NEU], lymphocyte count [LYM], and monocyte count [MONO]), and tumor-related information (tumor site, tumor regression grade [TRG], tumor stage, perineural invasion, and vessel invasion). Except for post neoadjuvant therapy pathological Tumor, Node, Metastasis [ypTNM] stage and TRG, all baseline information was based on the outcome of the first hospitalization.

### 2.3 Definition and construction of SII-PNI score

PNI and SII were defined as follows: SII = PLT (10^^9^/L) × NEU (10^^9^/L)/LYM (10^^9^/L); PNI = ALB (g/L) + 5 × LYM (10^^9^/L). Using the combined cohort (Combined from FJCH cohort, WHUH cohort, 900th Hospital cohort) data, we calculated the Youden index (sensitivity + specificity-1) maxima to determine the optimal cutoff values, The specific analysis process was as follows: First, the Youden index was calculated for all possible cutoff points using the R software (version 4.3.0). The value that maximized this index was selected as the optimal threshold. The results showed that the optimal cutoff value for SII was 593.590 (AUC = 0.679, 95% CI: 0.616-0.742), and the optimal cutoff value for PNI was 49.525 (AUC = 0.667, 95% CI: 0.598-0.737). We categorized SII and PNI into low SII, high SII, low PNI, and high PNI based on the optimal cutoff values. Based on this, patients were categorized into three groups: those with an SII-PNI score of 2 (high SII and high PNI), an SII-PNI score of 1 (either high SII and low PNI or low SII and high PNI), and an SII-PNI score of 0 (low SII and low PNI).

### 2.4 Follow-up and end points

Follow-up information was obtained through clinical consultation records, imaging data, and telephone follow-up visits, which continued until October 31, 2024 or the patient’s death, whichever occurred first. The primary outcome of this study was pCR, defined as surgical resection specimens that showed no tumor cell remnants on pathological examination (i.e., ypT_0_N_0_cM_0_). the secondary outcomes were DFS, defined as the time from surgery to disease recurrence or death from any cause (whichever occurred first), and OS, defined as the time from the start of nICT to death from any cause.

### 2.5 Treatment options

Most patients in this study received two to three cycles of neoadjuvant immunotherapy combined with chemotherapy before surgery. This regimen consisted of an intravenous infusion of an immune checkpoint inhibitor (camrelizumab 200 mg, tislelizumab 200 mg, sintilimab 200 mg, pembrolizumab 2 mg/kg, or nivolumab 3 mg/kg) on ​​day 1 of each cycle, combined with a paclitaxel- and platinum-based chemotherapy regimen (e.g., nab-paclitaxel 125 mg/m² administered intravenously on days 1 and 8 of each cycle, or paclitaxel 175 mg/m² administered intravenously on day 1 of each cycle combined with cisplatin 75 mg/m² administered intravenously on day 1 of each cycle). Curative resection, typically using an Ivor Lewis or McKeown esophagectomy, was performed 4 to 6 weeks after completion of neoadjuvant therapy. Postoperative adjuvant therapy is not mandatory and needs to be formulated according to the postoperative pathological results and the specific conditions of the patient. However, for patients with esophageal squamous cell carcinoma with pathological stage ypT_0-4a_N_+_M_0_, adjuvant therapy is recommended. For patients with ypT_0-4a_N_0_M_0_, adjuvant therapy is recommended based on close monitoring. Postoperative adjuvant therapy options include chemotherapy combined with immunotherapy, immunotherapy, chemotherapy.

### 2.6 Statistics

Continuous variables were expressed and statistically tested according to their data distribution characteristics. One-way ANOVA was used and expressed as mean ± standard deviation (Mean ± SD) if they were normally distributed and met the requirements to satisfy variance chi-square. If they did not meet the criteria for normal distribution, they were analyzed by the Kruskal-Wallis rank sum test and expressed as median and interquartile range (Median, IQR). Categorical variables were expressed as frequencies and proportions (n, %), and Pearson’s chi-square test or the continuity-corrected chi-square test was used. We used maximization of the Youden index to calculate the optimal thresholds for SII and PNI, ROC curves to assess the predictive performance of the SII-PNI scores. When performing univariate and multivariate logistic regression analyses of independent predictors of pCR, we performed multicollinearity diagnosis and calculated the variance inflation factor (VIF). The VIF of all variables was less than 4, indicating that there was no serious multicollinearity problem. Survival analyses were performed using the Kaplan-Meier method; overall and pairwise comparisons were performed using the log-rank test, and p-value correction was performed using the Bonferroni method when comparing differences in DFS and OS among the three patient groups. Univariate and multivariate Cox were used to estimate the hazard ratios for DFS and OS in the three patient groups. Multicollinearity was also diagnosed in the Cox regression analysis, and the VIFs for all variables were less than 4, confirming model stability. Predictors meeting the p-value threshold (*P* < 0.1) in univariate analysis were included in the multivariate Cox regression. In subgroup analyses, hazard ratios (HRs) for DFS and OS were calculated using non-stratified univariate Cox, and the results were presented as forest plots. Two-tailed tests were applied for all statistical analyses, with a significance level of *P* < 0.05. All statistical analyses were performed using SPSS software version 26.0 (IBM, Chicago, IL, USA) and R software version 4.3.0 (R Foundation) for data analysis.

## 3 Results

### 3.1 Patient characteristics

A total of 345 patients with ESCC treated with nICT were included in this retrospective multicenter cohort study, including 70 patients with an SII-PNI score of 0, 149 patients with a score of 1, and 126 patients with a score of 2. The baseline demographic characteristics of patients in the different SII-PNI score groups in the combined cohort are shown in **Table 1**. The different SII-PNI score groups differed in terms of gender (*P* = 0.005), BMI (*P* = 0.034), smoking (*P* = 0.002), drinking (*P* = 0.040), cTNM stage (*P* = 0.002), ypTNM stage (*P* < 0.001), and TRG (*P* < 0.001). Specifically, there were more patients with cTNM stage IV (38.9%), ypTNM stage III (43.7%) and IVA (7.9%), and TRG grade 3 (40.5%) tumors in the 2-score group. In contrast, there were fewer patients with cTNM stage IV tumors (22.9%), ypTNM stage III (14.3%) and IVA (2.9%), and TRG grade 3 (17.1%) in the 0-score group, with the 1-score group in between. In addition, we compared hematologic characteristics among the different SII-PNI score groups (**Supplementary Table 1**).

**Table 1:** Baseline characteristics of patients in the 0-score group, 1-score group, and 2-score group in the combined cohort.

Variables	SII-PNI score			*P* value
	0 score (n = 70)	1 score (n = 149)	2 score (n = 126)	
Sex, n (%)				0.005
Male	53 (75.7%)	110 (73.8%)	112 (88.9%)	
Female	17 (24.3%)	39 (26.2%)	14 (11.1%)	
Age (years), mean ± SD	62.14 ± 6.44	62.28 ± 7.47	61.71 ± 7.43	0.806
BMI (kg/m^2^), median (IQR)	22.92 (20.46, 24.61)	21.62 (20.07, 23.73)	21.94 (19.53, 23.62)	0.034
ECOG PS score, n (%)				0.151
0 score	35 (50.0%)	95 (63.8%)	76 (60.3%)	
1 score	35 (50.0%)	54 (36.2%)	50 (39.7%)	
Smoking, n (%)				0.002
No	45 (64.3%)	103 (69.1%)	61 (48.4%)	
Yes	25 (35.7%)	46 (30.9%)	65 (51.6%)	
Drinking, n (%)				0.040
No	48 (68.6%)	117 (78.5%)	82 (65.1%)	
Yes	22 (31.4%)	32 (21.5%)	44 (34.9%)	
Hypertension, n (%)				0.119
No	55 (78.6%)	126 (84.6%)	94 (74.6%)	
Yes	15 (21.4%)	23 (15.4%)	32 (25.4%)	
Tumor Location, n (%)				0.598
Upper thoracic	13 (18.6%)	27 (18.1%)	30 (23.8%)	
Middle thoracic	36 (51.4%)	72 (48.3%)	52 (41.3%)	
Lower thoracic	21 (30.0%)	50 (33.6%)	44 (34.9%)	
Vessel invasion, n (%)				0.252
No	53 (75.7%)	100 (67.1%)	81 (64.3%)	
Yes	17 (24.3%)	49 (32.9%)	45 (35.7%)	
Perineural invasion, n (%)				0.408
No	60 (85.7%)	117 (78.5%)	99 (78.6%)	
Yes	10 (14.3%)	32 (21.5%)	27 (21.4%)	
cTNM stage (AJCC 8th), n (%)				0.002
II	20 (28.6%)	15 (10.1%)	19 (15.1%)	
III	34 (48.6%)	90 (60.4%)	58 (46%)	
IV	16 (22.9%)	44 (29.5%)	49 (38.9%)	
ypT stage, n (%)				< 0.001
T0	51 (72.9%)	35 (23.5%)	19 (15.1%)	
T1	3 (4.3%)	26 (17.4%)	27 (21.4%)	
T2	4 (5.7%)	31 (20.8%)	18 (14.3%)	
T3	12 (17.1%)	53 (35.6%)	59 (46.0%)	
T4a	0 (0.0%)	4 (2.7%)	4 (3.2%)	
ypN stage, n (%)				< 0.001
N0	58 (82.9%)	89 (59.7%)	61 (48.4%)	
N1	5 (7.1%)	37 (24.8%)	47 (37.3%)	
N2	5 (7.1%)	19 (12.8%)	12 (9.5%)	
N3	2 (2.9%)	4 (2.7%)	6 (4.8%)	
ypTNM stage (AJCC 8th), n (%)				< 0.001
I	51 (72.9%)	66 (44.3%)	45 (35.7%)	
II	7 (10.0%)	21 (14.1%)	16 (12.7%)	
III	10 (14.3%)	54 (36.2%)	55 (43.7%)	
IV	2 (2.9%)	8 (5.4%)	10 (7.9%)	
TRG (AJCC 8th), n (%)				< 0.001
0	44 (62.9%)	29 (19.5%)	5 (4.0%)	
1	11 (15.7%)	42 (28.2%)	34 (27.0%)	
2	3 (4.3%)	27 (18.1%)	36 (28.6%)	
3	12 (17.1%)	51 (34.2%)	51 (40.5%)	
pCR, n (%)				< 0.001
non-pCR	26 (37.1%)	120 (80.5%)	121 (96.0%)	
pCR	44 (62.9%)	29 (19.5%)	5 (4.0%)	

For continuous variables: If data followed a normal distribution and met the chi-square test requirements, they were described using mean ± standard deviation (Mean ± SD). If not normally distributed, they were presented as median and interquartile range (Median, IQR). Categorical variables were expressed as frequencies and proportions (n, %). AJCC, American Joint Committee on Cancer; BMI, Body Mass Index; ECOG PS score, Eastern Cooperative Oncology Group Performance Status score; cTNM, Clinical Tumor Node Metastasis; ypTNM, postneoadjuvant therapy pathological Tumor, Node, Metastasis; TRG, Tumor Regression Grade; pCR, Pathological Complete Response.

### 3.2 Predictive performance of SII-PNI score

**Figure 1:**
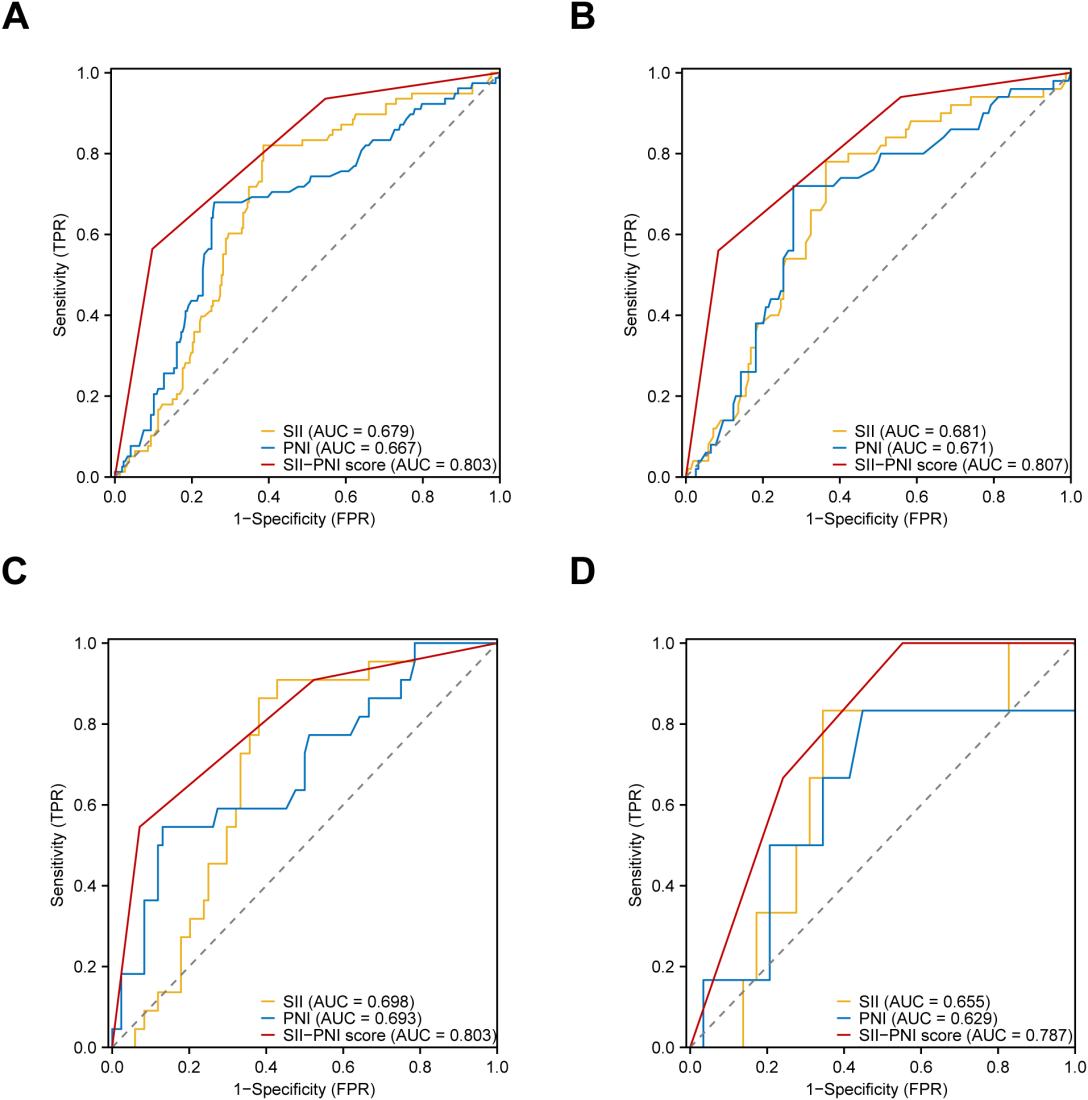
The predictive performance of the SII-PNI score and its individual components (SII and PNI) for pCR. **(A)** ROC curve of the combined cohort; **(B)** ROC curve of the FJCH cohort; **(C)** ROC curve of the WHUH cohort; **(D)** ROC curve of the 900th Hospital cohort. WHUH, Wuhan Union Hospital; FJCH, Fujian Provincial Cancer Hospital; 900th Hospital, The 900 Hospital of the Joint Service Support Force of the People’s Liberation Army of China; SII, Systemic Immune-Inflammation Index; PNI, Prognostic Nutritional Index; ROC, Receiver Operating Characteristic; AUC, Area Under the Receiver Operating Characteristic Curve.

The predictive efficacy of the SII-PNI score and its components (SII and PNI) for pCR after nICT was assessed using ROC curves in four cohorts (**Figure 1**). The DeLong’s test confirmed that the SII-PNI score had superior predictive power compared to its individual components. The statistical significance and specific AUC values are shown in **Supplementary Figure 1**. In the combined cohort (**Figure 1A**), the AUC of the SII-PNI score was 0.803 (95% CI: 0.753-0.854), and, compared to the SII-PNI score, SII (AUC = 0.679, 95% CI: 0.616-0.742, *P* < 0.001) and PNI (AUC = 0.667, 95% CI: 0.598-0.737, *P* < 0.001) had lower predictive efficacy. This trend remained stable in the other cohorts: the FJCH cohort (**Figure 1B**) (AUC: SII-PNI score = 0.807 [reference], SII = 0.681 [95% CI: 0.600-0.763, *P* < 0.001], PNI = 0.671 [95% CI: 0.586-0.757, *P* < 0.001]), the WHUH cohort (**Figure 1C**) (AUC: SII-PNI score = 0.803 [reference], SII = 0.698 [95% CI: 0.593-0.802, *P* = 0.012], PNI = 0.693 [95% CI: 0.563-0.823, *P* = 0.014]), and 900th Hospital cohort (**Figure 1D**) (AUC: SII-PNI score = 0.787 [reference], SII = 0.655 [95% CI: 0.419-0.891, *P* = 0.131], PNI = 0.629 [95% CI: 0.341-0.918, *P* = 0.141]). The trend that the predictive efficacy of SII-PNI score was higher than that of SII and PNI in all cohorts was consistent across centers, which suggests that the SII-PNI score has stable predictive performance and can be used as a reliable clinical tool for predicting pCR after nICT.

### 3.3 Logistic analysis and model evaluation

Univariate logistic regression analysis of pCR in the combined cohort, SII-PNI score, BMI, cTNM staging, vessel invasion, and perineural invasion were identified as potential predictors of pCR and were subsequently included in multivariate analysis. Multivariate logistic regression analysis revealed that the SII-PNI score, BMI, cTNM stage, and vessel invasion were independent predictors of pCR. Specifically, Compared to the 0-score group, patients in the 1-score group had a lower pCR rate (OR = 0.159, 95% CI:0.080-0.319, *P* < 0.001), while those in the 2-score group showed an even lower pCR rate (OR = 0.025, 95% CI:0.009-0.073, *P* < 0.001). Additionally, in cTNM staging, stage III patients had a lower pCR rate compared to stage II patients (OR = 0.397, 95% CI:0.171-0.919, *P* = 0.031), and stage IVA patients exhibited a further reduction in pCR rate (OR = 0.250, 95% CI:0.094-0.664, *P* = 0.005). An increase in BMI was significantly associated with a higher pCR rate (OR = 1.145, 95% CI:1.019-1.287, *P* = 0.023). Patients with positive vessel invasion had a reduced pCR rate (OR = 0.414, 95% CI:0.177-0.968, *P* = 0.042). Multicollinearity diagnosis confirmed that the VIF of all variables in the multivariate logistic regression was less than 4, indicating that there was no serious multicollinearity, ensuring the stability of the model estimates (**Table 2**).

**Table 2:** Univariate and multivariate logistic regression analyses of pCR in the combined cohort.

Characteristics	Univariate analysis		Multivariate analysis		
	OR (95% CI)	*P* value	OR (95% CI)	*P* value	VIF
SII-PNI score					
0 score	Reference		Reference		
1 score	0.143 (0.076 - 0.269)	< 0.001	0.159 (0.080 - 0.319)	< 0.001	1.211
2 score	0.024 (0.009 - 0.068)	< 0.001	0.025 (0.009 - 0.073)	< 0.001	1.214
Sex					
Female	Reference				
Male	0.806 (0.439 - 1.480)	0.487			
Age (years)	0.983 (0.950 - 1.018)	0.341			
BMI (kg/m^2^)	1.169 (1.068 - 1.279)	< 0.001	1.145 (1.019 - 1.287)	0.023	1.017
ECOG PS score					
0 score	Reference				
1 score	1.276 (0.766 - 2.123)	0.349			
Smoking					
No	Reference				
Yes	0.824 (0.489 - 1.391)	0.470			
Drinking					
No	Reference				
Yes	1.071 (0.615 - 1.865)	0.810			
cTNM stage (AJCC 8th)					
II	Reference		Reference		
III	0.325 (0.171 - 0.619)	< 0.001	0.397 (0.171 - 0.919)	0.031	1.748
IV	0.164 (0.076 - 0.356)	< 0.001	0.250 (0.094 - 0.664)	0.005	1.733
Hypertension					
No	Reference				
Yes	0.824 (0.431 - 1.577)	0.559			
Vessel invasion					
No	Reference		Reference		
Yes	0.274 (0.138 - 0.543)	< 0.001	0.414 (0.177 - 0.968)	0.042	1.170
Perineural invasion					
No	Reference		Reference		
Yes	0.217 (0.084 - 0.561)	0.002	0.332 (0.108 - 1.019)	0.054	1.133
Tumor Location					
Upper thoracic	Reference				
Middle thoracic	1.611 (0.803 - 3.234)	0.179			
Lower thoracic	1.037 (0.485 - 2.220)	0.925			
CEA	0.934 (0.799 - 1.092)	0.391			

The SII-PNI score was an independent predictor of pCR. CI, confidence interval; OR, Odds ratio; BMI, Body Mass Index; ECOG PS score, Eastern Cooperative Oncology Group Performance Status score; AJCC, American Joint Committee on Cancer; cTNM, Clinical Tumor Node Metastasis; CEA, Carcinoembryonic Antigen; pCR, Pathological Complete Response; VIF, variance inflation factor.

**Figure 2:**
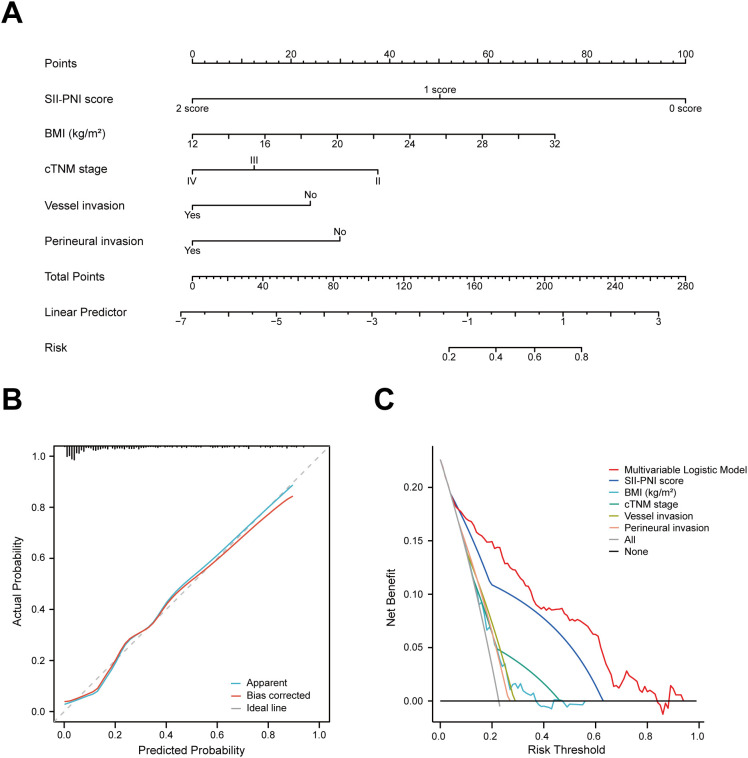
**(A)** Nomogram for pCR, developed based on multivariable logistic regression analysis. **(B)** Calibration curves of the Multivariable Logistic Model. The three calibration curves nearly overlap, indicating high reliability of the model predictions. **(C)** Decision curve analysis. The x-axis represents the risk probability threshold, and the y-axis indicates the net benefit rate. “All”, intervention for the entire population; “None”, no intervention; cTNM, Clinical Tumor Node Metastasis; BMI, Body Mass Index; pCR, Pathological Complete Response.

The nomogram constructed based on independent predictors identified through multivariate logistic regression demonstrated reliable predictive performance (**Figure 2A**). Both the predicted probability curve and calibration curve closely aligned with the ideal reference line, indicating that the model-predicted probabilities closely matched the actual observed values, confirming the model’s good accuracy (**Figure 2B**). Decision curve analysis (DCA) further validated the clinical utility of the integrated model and SII-PNI score: the multivariate logistic model showed higher net benefit than other clinical indicators and the “All” and “None” reference lines within the 10%-80% risk threshold range, while the SII-PNI score outperformed other clinical indicators and the reference lines within the 10%-60% risk threshold range, indicating that the net benefits of the integrated model and SII-PNI score were significantly higher than those of other clinical indicators (**Figure 2C**).

### 3.4 Survival analysis

**Figure 3:**
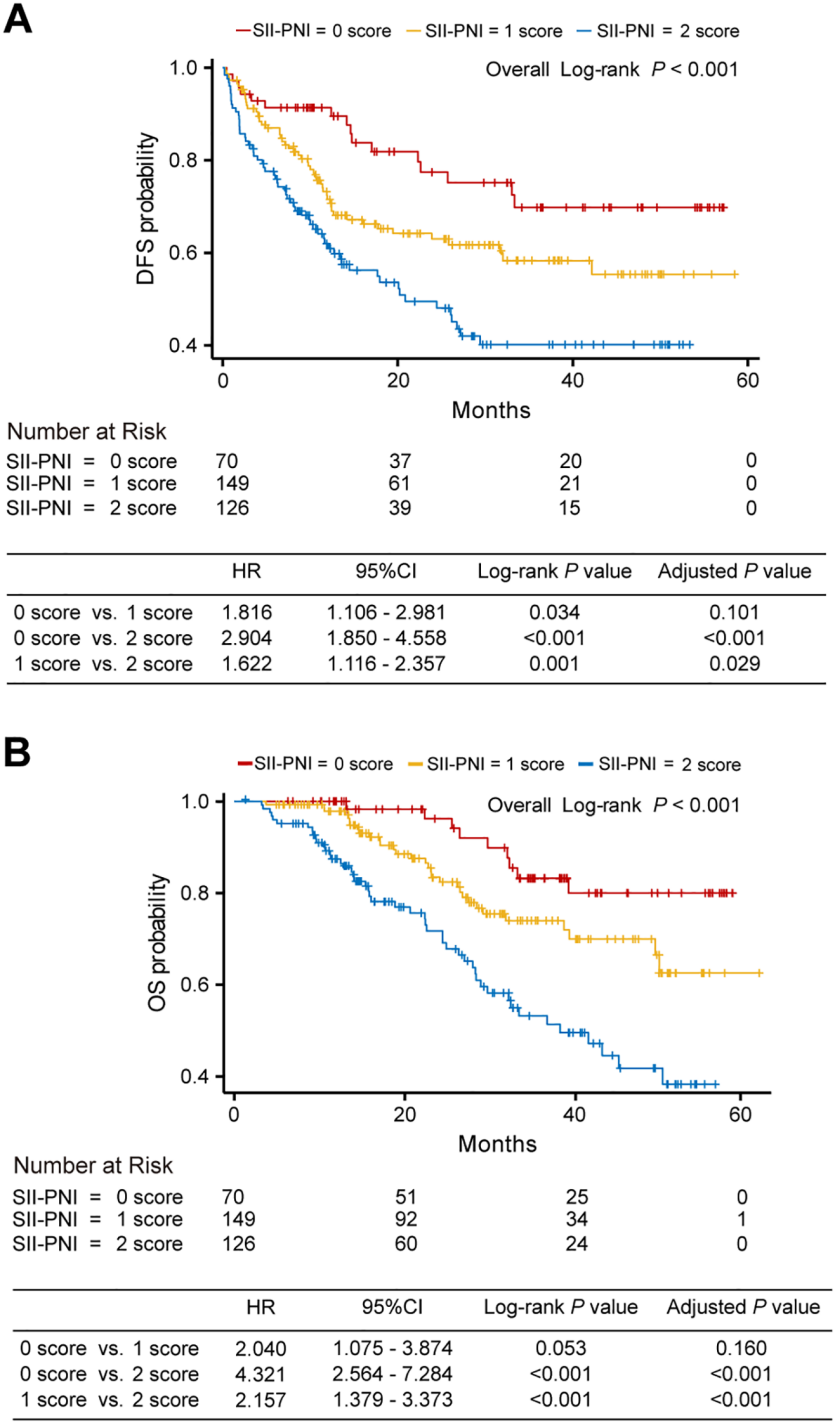
Kaplan-Meyer curves of DFS **(A)** and OS **(B)** for subgroup 0 (red), subgroup 1 (yellow), and subgroup 2 (blue) in the combined cohort. DFS, disease-free survival; OS, overall survival; CI, confidence interval; HR, Hazard ratio.

The median follow-up time was 25.66 months (interquartile range [IQR]: 14.03-39.26 months; IQR = 25.23 months), 9 of 70 patients (12.9%) in the 0-score group, 31 of 149 patients (20.8%) in the 1-score group, and 48 of 126 patients (38.1%) in the 2-score group died. Significant differences in OS (overall log-rank *P* < 0.001) and DFS (overall log-rank *P* < 0.001) were found in the combined cohort for each SII-PNI score group. Specifically, compared with the 0-score group, the 1-score and 2-score groups showed higher risks for both DFS and OS. For DFS (**Figure 3A**), compared with the 0-score group, the 1-score group had an elevated risk (HR = 1.816, 95% CI:1.106-2.981, adjusted *P* = 0.101), and the 2-score group showed a further increased risk (HR = 2.904, 95% CI:1.850-4.558, adjusted *P* < 0.001). Compared with the 1-score group, the 2-score group also exhibited a higher risk (HR = 1.622, 95% CI:1.116-2.357, adjusted *P* = 0.029). For OS (**Figure 3B**), compared with the 0-score group, the 1-score group demonstrated an elevated risk (HR = 2.040, 95% CI:1.075-3.874, adjusted *P* = 0.160), and the 2-score group displayed a further increased risk (HR = 4.321, 95% CI:2.564-7.284, adjusted *P* < 0.001). Compared with the 1-score group, the 2-score group was associated with a similarly elevated risk (HR = 2.157, 95% CI:1.379-3.373, adjusted *P* < 0.001). The median DFS and OS in the 2-score group were 20.86 months and 38.17 months, respectively. Due to insufficient follow-up, the median DFS and OS could not be determined for the 0-score and 1-score groups. We also constructed Kaplan-Meier curves for DFS and OS in the FJCH cohort, WHUH cohort, and 900th Hospital cohort, respectively, which yielded results consistent with those in the integrated cohort (**Supplementary Figure 2**).

### 3.5 Cox regression analysis and subgroup analysis

In univariate Cox proportional hazards regression analysis of OS in the combined cohort, the SII-PNI score, ECOG PS score, cTNM stage, vessel invasion, and perineural invasion were identified as potential predictors and included in the multivariate analysis. In the multivariate Cox proportional hazards regression analysis, independent risk factors for OS included a poorer SII-PNI score (1-score group: HR = 2.169, 95% CI:1.006-4.676, *P* = 0.048; 2-score group: HR = 4.473, 95% CI:2.138-9.357, *P* < 0.001) and ECOG PS score (HR = 1.699, 95% CI:1.109-2.602, *P* = 0.015), both of which were significantly associated with reduced OS (**Table 3**). Similarly, in univariate Cox proportional hazards regression analysis of DFS in the combined cohort, the SII-PNI score, cTNM stage, vascular invasion, and perineural invasion were identified as potential predictors and included in the multivariate analysis. 2-score group (HR = 2.487, 95% CI:1.414-4.374, *P* = 0.002), cTNM stage IV (HR = 2.010, 95% CI:1.005-4.021, *P* = 0.048), and perineural invasion (HR = 1.607, 95% CI:1.052-2.455, *P* = 0.028) emerged as independent risk factors for reduced DFS (**Supplementary Table 2**). For both multivariate Cox proportional hazards regression analysis (OS and DFS), the VIF for all included variables were below 4, confirming the absence of severe multicollinearity and the stability of the estimates.

**Table 3:** Univariate and multivariate Cox proportional hazards analyses of OS in the combined cohort.

Characteristics	Univariate analysis		Multivariate analysis		
	HR (95% CI)	*P* value	HR (95% CI)	*P* value	VIF
SII-PNI score					
0 score	Reference		Reference		
1 score	2.041 (0.971 - 4.293)	0.060	2.169 (1.006 - 4.676)	0.048	3.079
2 score	4.414 (2.162 - 9.012)	< 0.001	4.473 (2.138 - 9.357)	< 0.001	3.086
Sex					
Female	Reference				
Male	1.151 (0.686 - 1.933)	0.594			
Age (years)	1.020 (0.990 - 1.051)	0.196			
BMI (kg/m^2^)	0.953 (0.886 - 1.025)	0.196			
ECOG PS score					
0 score	Reference		Reference		
1 score	1.536 (1.008 - 2.341)	0.046	1.699 (1.109 - 2.602)	0.015	1.026
Smoking					
No	Reference				
Yes	1.129 (0.738 - 1.727)	0.577			
Drinking					
No	Reference				
Yes	0.974 (0.612 - 1.550)	0.912			
cTNM stage (AJCC 8th)					
II	Reference		Reference		
III	1.190 (0.551 - 2.573)	0.658	0.778 (0.349 - 1.733)	0.539	3.474
IV	2.040 (0.962 - 4.326)	0.063	1.199 (0.538 - 2.673)	0.658	3.656
Hypertension					
No	Reference				
Yes	1.246 (0.750 - 2.071)	0.396			
Vessel invasion					
No	Reference		Reference		
Yes	1.559 (1.022 - 2.379)	0.039	1.214 (0.763 - 1.930)	0.413	1.205
Perineural invasion					
No	Reference		Reference		
Yes	1.616 (0.989 - 2.640)	0.056	1.529 (0.908 - 2.574)	0.110	1.122
Tumor Location					
Upper thoracic	Reference				
Middle thoracic	0.741 (0.444 - 1.235)	0.250			
Lower thoracic	0.590 (0.324 - 1.073)	0.084			
CEA	1.005 (0.974 - 1.037)	0.766			

The SII-PNI score demonstrated significant prognostic stratification ability for OS. OS, Overall Survival; CI, confidence interval; HR, Hazard ratio; BMI, Body Mass Index; ECOG PS score, Eastern Cooperative Oncology Group Performance Status score; AJCC, American Joint Committee on Cancer; cTNM, Clinical Tumor Node Metastasis; CEA, Carcinoembryonic Antigen; VIF, variance inflation factor.

**Figure 4:**
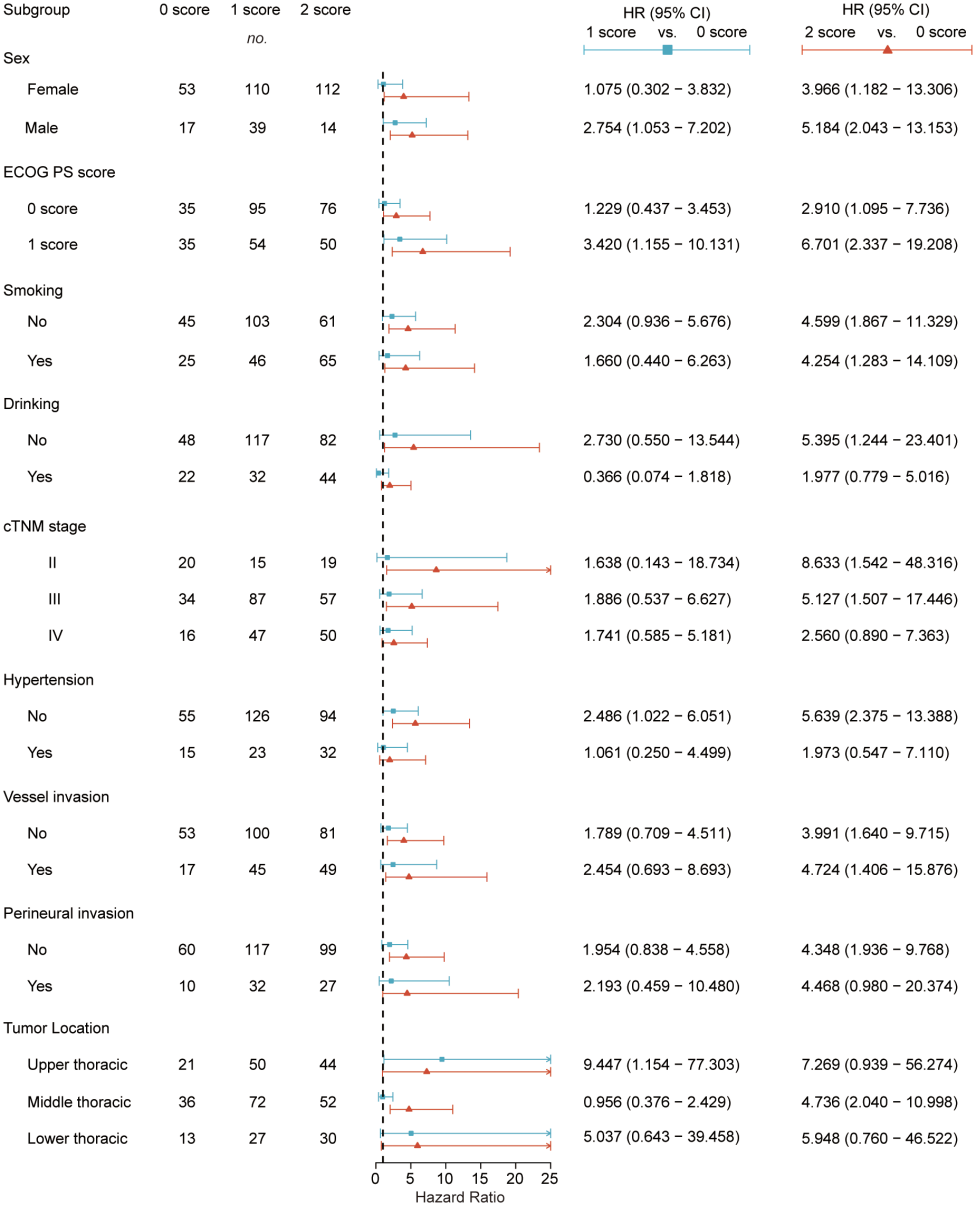
Subgroup analysis of OS in the 0-score, 1-score, and 2-score groups in the combined cohort. Hazard ratios were derived from univariate Cox models for each subgroup. The dashed line indicates a hazard ratio of 1. 1-score group vs. 0-score group (blue), 2-score group vs. 0-score group (red). OS, Overall Survival; CI, confidence interval; HR, Hazard ratio; ECOG PS score, Eastern Cooperative Oncology Group Performance Status score; cTNM, Clinical Tumor Node Metastasis.

To clarify the association between SII-PNI score and OS/DFS risk across baseline characteristics (including gender, ECOG PS score, smoking history, alcohol history, clinical TNM stage, hypertension, vascular invasion, perineural invasion, and tumor location), We performed subgroup analyses in the combined cohort. Results demonstrated that, in most subgroups, patients with poorer SII-PNI scores (1-score group and 2-score group) showed an increasing trend in mortality risk for OS (**Figure 4**) and disease progression risk for DFS (**Supplementary Figure 3**) compared to 0-score group, with risk exhibiting a graded dependence on score severity (higher risk in 2-score group than in 1-score group).

## 4 Discussion

In this study, the SII-PNI score was constructed by integrating LYM, ALB, NEU, and PLT data, and the SII-PNI score was effective in predicting the pCR rate and long-term prognosis of ESCC patients treated with nICT. Multicenter validation (combined cohort, FJCH cohort, WHUH cohort, and 900th Hospital cohort) confirmed its robust predictive performance and prognostic stratification value. In addition, we demonstrated that the SII-PNI score was an independent predictor of pCR, OS, and DFS by logistic regression and Cox proportional hazards regression analyses. Overall, these findings increase the number of biomarkers available for predicting the efficacy of nICT and provide clinical guidance for ESCC patients treated with nICT.

In recent years, immunotherapy combined with chemotherapy has become a new direction in the treatment of esophageal cancer. Immune checkpoint inhibitors, centered on PD-1/PD-L1 inhibitors, inhibit immune checkpoints to downregulate immune responses and enhance anti-tumor immunity by blocking the interaction between immune checkpoints and T lymphocytes (20, 21). Immunotherapeutic agents and chemotherapeutic agents can work together to regulate the tumor microenvironment, significantly increase the immune checkpoint inhibitor response rate, and achieve synergistic anti-tumor effects (22). Data from the phase III clinical trial ESCORT-NEO/NCCES 01 showed that the pCR rate in the neoadjuvant immunotherapy combined with chemotherapy group was 28.0%, which was significantly higher than that of the chemotherapy-only group (4.7%), suggesting that neoadjuvant immunotherapy combined with chemotherapy can enhance the pCR rate [9]. In another study, Ruan et al. (23) retrospectively collected data from 192 patients with LA-ESCC treated with nICT and performed Kaplan-Meier survival analysis on the pCR and non-pCR groups, which showed that the RFS and OS of pCR patients were significantly better than those of non-pCR patients (2-year RFS: 86% *vs* 61%; 2-year OS: 95% *vs* 75%). Although pCR is a key survival predictor after neoadjuvant therapy (24, 25), there is still a lack of relatively reliable and economical predictors of pCR.

The cutoff values for the SII and PNI identified in this study (593.590 and 49.525, respectively) demonstrate strong concordance with previously reported ranges in the ESCC literature, thereby affirming their reliability. Notably, our SII value closely aligns with the 559.266 reported by Zhang et al. (15) in a comparable nICT cohort. Similarly, the PNI value is highly comparable to the 48.35 and 53.585 reported by Li et al. (26) and Song et al. (27), respectively. These cutoff values fall within the broadly accepted clinical range, and their predictive efficacy has been rigorously validated in our multicenter cohort, underscoring their potential applicability in clinical practice. To our knowledge, this is the first study to report the relationship between the SII-PNI score and pCR/prognosis in ESCC patients treated with nICT. Our results showed that SII-PNI score predicted pCR after nICT with an AUC of 0.803, and poorer scores (group 1/2) were associated with shorter DFS and OS. In another cancer, Ding et al. (28). reported that in patients with locally advanced gastric cancer receiving neoadjuvant chemotherapy, the ORR was highest in 0-score group and lowest in 2-score group (*P* < 0.001), and SII-PNI score was an independent predictor of OS (HR = 4.982, 95% CI: 1.890-10.234, *P* = 0.001) and DFS (HR = 4.763, 95% CI: 1.994-13.903, *P* = 0.001), which is similar to our results. In addition, Feng et al. (29). integrated BMI, NEUT, neutrophil-lymphocyte ratio (NLR), lymphocyte-monocyte ratio (LMR), HB, C-reactive protein-albumin ratio (CAR), PLT, and hemoglobin-albumin-lymphocyte-platelet index (HALP) to construct the Integrated Inflammatory Nutritional Score (IINS). However, the IINS had limited predictive effect (total cohort AUC = 0.68, training set AUC = 0.70, validation set AUC = 0.66), and they did not perform a prognostic analysis, which is important for cancer research. In another study, Han et al. (30) conducted a retrospective study of 97 ESCC patients treated with nICT. Specifically, they constructed a nomogram model to predict the pCR rate by combining SII and clinical staging. Although the predictive efficacy was fair (AUC = 0.72 for the training set and 0.82 for the validation set), the predictive probabilities of the model do not match the actual risk, and the net return is low. In contrast, this study, based on the advanced concept of multi-source data integration (31), constructed the SII-PNI score by combining LYM, ALB, NEU, and PLT, which can more comprehensively reflect the overall nutritional and inflammatory status of the body. In the combined cohort, the SII-PNI score demonstrated excellent predictive performance and prognostic stratification capability. We validated the predictive performance of the SII-PNI score in three independent cohorts, all of which demonstrated good predictive performance (AUC: FJCH cohort = 0.807, WHUH cohort = 0.803, 900th Hospital cohort = 0.787). The SII-PNI score outperformed SII and PNI in predictive performance, except in the 900th Hospital cohort (all DeLong tests *P* < 0.05). In the 900th Hospital cohort, the AUC difference between the SII-PNI score and SII or PNI did not reach statistical significance, which may be due to the small sample size and insufficient statistical power, resulting in effects that exist but do not reach statistical significance. Notably, the superior predictive performance of the SII-PNI score compared to SII and PNI was consistent across all cohorts, indicating that the SII-PNI score has stable predictive performance and can serve as a reliable clinical tool for predicting pCR after nICT.

The SII-PNI score demonstrates good predictive value for pCR and prognostic stratification, and its underlying mechanism may involve the roles of LYM, ALB, NEU, and PLT in tumor progression. During cellular immunity, LYM acts as a major effector cell. It inhibits tumor proliferation, migration, and invasion through multidimensional mechanisms such as cytotoxic responses (32, 33). Decreased LYM is usually associated with poor prognosis and impaired immune surveillance, which can promote tumor progression (34, 35). ALB, a key nutritional marker, reflects metabolic status, with hypoalbuminemia linked to increased mortality (36, 37). Notably, NEU synergistically promotes tumor progression: In the tumor microenvironment (TME), cytokines (TGF-β, IL-8, IL-6, IL-17) drive NEU polarization into the N2 phenotype. These N2 NEU secrete pro-angiogenic factors (e.g., VEGF) and matrix metalloproteinases (MMPs) to enhance tumor growth and metastasis. Additionally, G-CSF and TGF-β in TME activate the ARG1 pathway in NEU, suppressing CD8+ T-cell proliferation and effector function (38–40). Furthermore, tumor cells upregulate PLT production via the IL-6-TPO axis. Activated PLT and tumor cells form an ADP-P2Y12 bidirectional loop. Through contact-independent and contact-dependent interactions, PLT-tumor interplay confers tumor-specific surface markers, creates a protective shield for immune evasion, and releases granule contents/exosomes to stimulate tumor proliferation (41, 42). This multicomponent synergy underscores that SII-PNI score systematically quantifies tumor-host interactions, offering a molecular basis for prognosis assessment.

This study has several limitations. First, this study was retrospective in design and had limited sample sizes in some subgroups, which may have led to insufficient statistical power. Therefore, future work should first focus on collecting more samples, especially expanding the cohort size in currently underrepresented subgroups, and then conducting prospective, multicenter studies to ultimately validate the findings. Second, the nomogram lacks fully independent external validation, which increases the potential risk of model overfitting. Its generalizability needs to be further confirmed in future independent cohorts. Third, this study was based on a static baseline SII-PNI score, failing to reveal the dynamic predictive value of this indicator during treatment. Future studies are warranted to continuously monitor the SII-PNI score throughout neoadjuvant therapy, which may provide dynamic insights into treatment response and long-term survival. Finally, the current model does not integrate important immune biomarkers such as PD-L1 and TMB. To further improve the overall predictive power, subsequent studies should focus on developing a composite model that combines the SII-PNI score with these key molecular features. Despite the limitations of the current analysis, we report for the first time the relationship between baseline SII-PNI score and pCR and survival outcomes in ESCC patients treated with nICT.

## 5 Conclusion

Our study demonstrates that the SII-PNI score effectively predicts pCR and survival outcomes in ESCC patients receiving nICT. Therefore, SII-PNI score should be routinely assessed in clinical practice to enable early identification of patients likely to benefit from nICT, advancing precision medicine in this population.

## Data Availability

The data analyzed in this study is subject to the following licenses/restrictions: The datasets used and/or analysed during the current study are available from the corresponding author on reasonable request. Requests to access these datasets should be directed to LY, yanglian@hust.edu.cn.
