# Supplementary material for: The crossroads of inflammation and nutrition: predicting neoadjuvant immunochemotherapy efficacy in esophageal squamous cell carcinoma patients
Source: Front Immunol. 2025 Nov 20;16:1663268. doi: 10.3389/fimmu.2025.1663268 (PMC12675481; doi:10.3389/fimmu.2025.1663268)
Supplement: Supplementary file 1 [file Table1.docx]

Supplementary Material

**Table of Contents**

[Supplementary Tables 2](#_Toc211097784)

[Supplementary Table 1. Blood characteristics of patients in the 0-score group, 1-score group, and 2-score group in the combined cohort 2](#_Toc211097785)

[Supplementary Table 2. Univariate and multivariate Cox proportional hazards analyses of DFS in the combined cohort 3](#_Toc211097786)

[Supplementary Figures and Figure legends 4](#_Toc211097787)

[Supplementary Figure 1 Bar Chart Comparison of AUC Values for Three Predictive Models Across Four Cohorts 4](#_Toc211097788)

[Supplementary Figure 2 Kaplan-Meier curves for DFS and OS stratified by SII-PNI score groups 5](#_Toc211097789)

[Supplementary Figure 3 Subgroup analysis of DFS in the combined cohort 6](#_Toc211097790)

Supplementary Tables

Supplementary Table 1. Blood characteristics of patients in the 0-score group, 1-score group, and 2-score group in the combined cohort

| Variables | SII-PNI score | | | *P* value |
| --- | --- | --- | --- | --- |
|  | 0 score (n = 70) | 1 score (n = 149) | 2 score (n = 126) |  |
| CEA, median (IQR) | 1.70 (1.18, 2.80) | 2 (1.30, 3.29) | 2.30 (1.49, 3.53) | 0.037 |
| TBIL, median (IQR) | 13.05 (11.33, 14.90) | 13.45 (10, 16.28) | 12.15 (9.28, 14.35) | 0.087 |
| ALT, median (IQR) | 14 (10.85, 18) | 14 (10, 18) | 13 (9, 16) | 0.053 |
| AST, median (IQR) | 19 (17, 21) | 19.25 (16, 22) | 17 (14, 20.13) | 0.002 |
| ALP, median (IQR) | 74.50 (63, 92) | 76.50 (66, 93.75) | 80 (68, 93.25) | 0.260 |
| ALB, median (IQR) | 42.75 (41.13, 44.28) | 40 (38.20, 42.80) | 39.20 (36.95,40.80) | < 0.001 |
| GLOB, median (IQR) | 28.25 (25.45, 32.38) | 29.25 (25.05,32.03) | 28.60 (26.60,31.45) | 0.927 |
| CRE, median (IQR) | 77 (69, 85.75) | 75 (65, 85) | 76 (65, 86) | 0.467 |
| BUN, median (IQR) | 4.63 (3.63, 5.61) | 4.71 (3.80, 5.66) | 4.50 (3.45, 5.58) | 0.486 |
| FBG, median (IQR) | 5.30 (4.96, 5.85) | 5.21 (4.83, 5.74) | 5.20 (4.70, 5.90) | 0.496 |
| TC, median (IQR) | 5.40 (4.50, 5.92) | 5.12 (4.35, 5.77) | 4.92 (3.95, 5.55) | 0.112 |
| TG, median (IQR) | 1.10 (0.94, 1.32) | 1.10 (0.85, 1.34) | 1.07 (0.85, 1.34) | 0.841 |
| HDL-C, median (IQR) | 1.28 (1.09, 1.48) | 1.19 (1.04, 1.42) | 1.18 (0.96, 1.31) | 0.013 |
| LDL-C, median (IQR) | 3.47 (2.79, 4.01) | 3.21 (2.56, 3.84) | 3.07 (2.36, 3.68) | 0.046 |
| LDH, median (IQR) | 180.50 (158.75,198.25) | 172 (156, 193) | 170 (155.25,191.50) | 0.139 |
| Ca, mean ± SD | 2.38 ± 0.13 | 2.34 ± 0.13 | 2.31 ± 0.13 | 0.00 3 |
| WBC, median (IQR) | 6.41 (5.49, 8.33) | 6.20 (5.01, 7.70) | 6.83 (5.80, 8.28) | 0.012 |
| HGB, median (IQR) | 139 (129.50, 146) | 132 (126, 144) | 134 (120.25,142.75) | 0.049 |
| PLT, median (IQR) | 229 (188, 257.50) | 217 (176, 272) | 266 (238, 340.75) | < 0.001 |
| NEU, median (IQR) | 3.70 (2.84, 4.21) | 3.60 (2.66, 4.89) | 4.90 (4.04, 6.08) | < 0.001 |
| LYM, median (IQR) | 2 (1.72, 2.46) | 1.70 (1.26, 2) | 1.50 (1.20, 1.76) | < 0.001 |
| MONO, median (IQR) | 0.38 (0.28, 0.56) | 0.43 (0.34, 0.51) | 0.47 (0.38, 0.60) | 0.003 |
| SII, median (IQR) | 404.20 (304.57,480.67) | 483 (361.31,659.37) | 891.65 (666.4,1295.3) | < 0.001 |
| PNI, median (IQR) | 52.83 (50.70, 55.46) | 48.50 (46.30,50.80) | 47.45 (44.30,48.60) | < 0.001 |

For continuous variables: If data followed a normal distribution and met the chi-square test requirements, they were described using mean ± standard deviation (Mean ± SD). If not normally distributed, they were presented as median and interquartile range (Median, IQR). carcinoembryonic antigen (CEA, ng/mL); total bilirubin (TBIL, μmol/L); alanine aminotransferase (ALT, U/L); aspartate aminotransferase (AST, U/L); alkaline phosphatase (ALP, U/L); albumin (ALB, g/L); globulin (GLOB, g/L); creatinine (CRE, μmol/L); urea nitrogen (BUN, mmol/L); fasting blood glucose (FBG, mmol/L); total cholesterol (TC, mmol/L); triglycerides (TG, mmol/L); high-density lipoprotein cholesterol (HDL-C, mmol/L); low-density lipoprotein cholesterol (LDL-C, mmol/L); lactate dehydrogenase (LDH, U/L); calcium (Ca, mmol/L); white blood cell count (WBC, 10⁹/L); hemoglobin (HGB, g/L); platelet count (PLT, 10⁹/L); neutrophil count (NEU, 10⁹/L); lymphocyte count (LYM, 10⁹/L); monocyte count (MONO, 10⁹/L).

Supplementary Table 2. Univariate and multivariate Cox proportional hazards analyses of DFS in the combined cohort

| Characteristics | Univariate analysis | | Multivariate analysis | | |
| --- | --- | --- | --- | --- | --- |
|  | HR (95% CI) | *P* value | HR (95% CI) | *P* value | VIF |
| SII-PNI score |  |  |  |  |  |
| 0 score | Reference |  | Reference |  |  |
| 1 score | 1.804 (1.029 - 3.161) | 0.039 | 1.548 (0.874 - 2.743) | 0.134 | 2.640 |
| 2 score | 2.947 (1.696 - 5.120) | < 0.001 | 2.487 (1.414 - 4.374) | 0.002 | 2.657 |
| Sex |  |  |  |  |  |
| Female | Reference |  |  |  |  |
| Male | 0.984 (0.644 - 1.505) | 0.942 |  |  |  |
| Age (years) | 1.012 (0.987 - 1.037) | 0.351 |  |  |  |
| BMI (kg/m^2^) | 0.984 (0.925 - 1.048) | 0.620 |  |  |  |
| ECOG PS score |  |  |  |  |  |
| 0 score | Reference |  |  |  |  |
| 1 score | 1.313 (0.929 - 1.855) | 0.123 |  |  |  |
| Smoking |  |  |  |  |  |
| No | Reference |  |  |  |  |
| Yes | 1.336 (0.944 - 1.891) | 0.102 |  |  |  |
| Drinking |  |  |  |  |  |
| No | Reference |  |  |  |  |
| Yes | 1.288 (0.893 - 1.858) | 0.175 |  |  |  |
| cTNM stage |  |  |  |  |  |
| Ⅱ | Reference |  | Reference |  |  |
| Ⅲ | 1.808 (0.926 - 3.530) | 0.083 | 1.505 (0.761 - 2.977) | 0.240 | 3.878 |
| Ⅳ | 2.794 (1.427 - 5.469) | 0.003 | 2.010 (1.005 - 4.021) | 0.048 | 3.962 |
| Hypertension |  |  |  |  |  |
| No | Reference |  |  |  |  |
| Yes | 1.344 (0.884 - 2.044) | 0.167 |  |  |  |
| Vessel invasion |  |  |  |  |  |
| No | Reference |  | Reference |  |  |
| Yes | 1.753 (1.236 - 2.486) | 0.002 | 1.358 (0.930 - 1.984) | 0.113 | 1.176 |
| Perineural invasion |  |  |  |  |  |
| No | Reference |  | Reference |  |  |
| Yes | 1.812 (1.223 - 2.685) | 0.003 | 1.607 (1.052 - 2.455) | 0.028 | 1.157 |
| Tumor Location |  |  |  |  |  |
| Upper thoracic | Reference |  |  |  |  |
| Middle thoracic | 0.829 (0.533 - 1.289) | 0.405 |  |  |  |
| Lower thoracic | 0.740 (0.455 - 1.203) | 0.224 |  |  |  |
| CEA | 1.001 (0.981 - 1.022) | 0.896 |  |  |  |

The SII-PNI score demonstrated significant prognostic stratification ability for DFS. DFS, Disease-Free Survival; CI, confidence interval; HR, Hazard ratio; BMI, Body Mass Index; ECOG PS score, Eastern Cooperative Oncology Group Performance Status score; cTNM, Clinical Tumor Node Metastasis; CEA, Carcinoembryonic Antigen; VIF, variance inflation factor.

Supplementary Figures and Figure legends

Supplementary Figure 1 Bar Chart Comparison of AUC Values for Three Predictive Models Across Four Cohorts


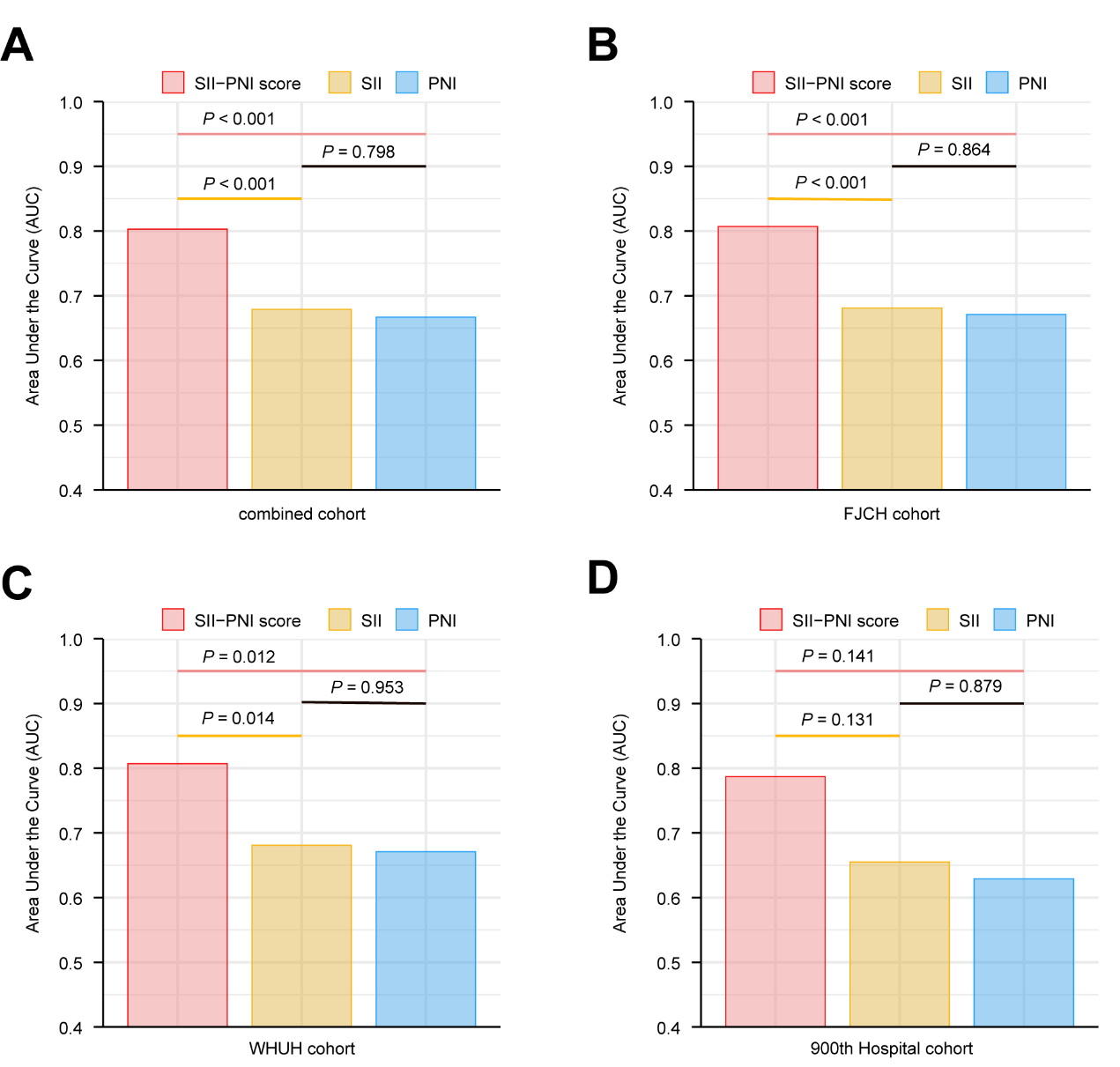


**Supplementary Figure 1** (A) Combined cohort, (B) FJCH cohort, (C) WHUH cohort, (D) 900th Hospital cohort. SII-PNI score (red), SII (yellow), PNI (blue), Bar height corresponds to AUC values of the indicators, all comparisons were performed using DeLong’s test.

Supplementary Figure 2 Kaplan-Meier curves for DFS and OS stratified by SII-PNI score groups

**
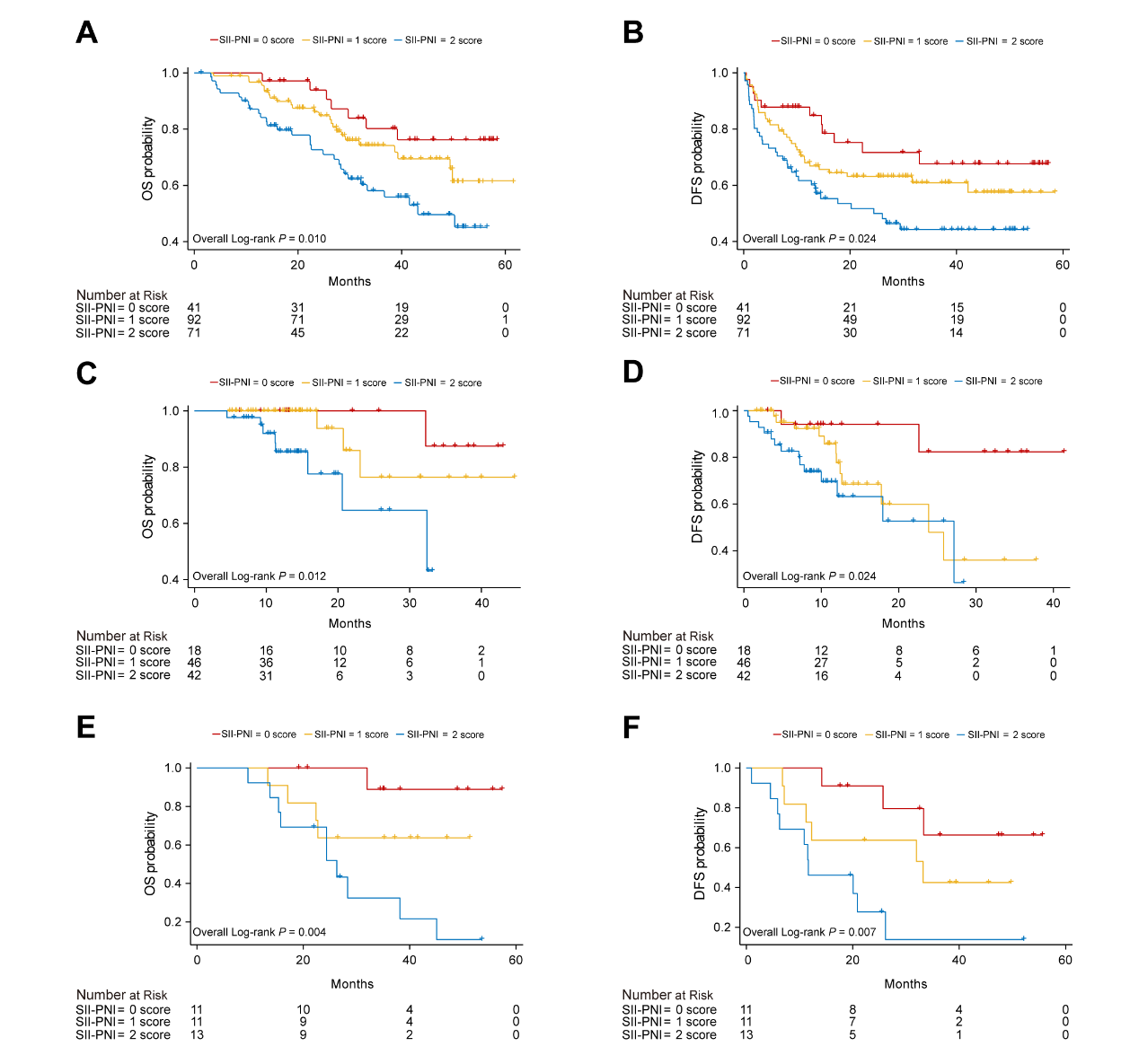
**

**Supplementary Figure 2** Kaplan-Meier curves for DFS and OS of the 0-score group (red), 1-score group (yellow), and 2-score group (blue). (A) Kaplan-Meier survival analysis of OS in the WHUH cohort; (B) Kaplan-Meier survival analysis of DFS in the WHUH cohort; (C) Kaplan-Meier survival analysis of OS in the FJCH cohort; (D) Kaplan-Meier survival analysis of DFS in the FJCH cohort; (E) Kaplan-Meier survival analysis of OS in 900th Hospital cohort; (F) Kaplan-Meier survival analysis of DFS in 900th Hospital cohort. DFS: disease-free survival; OS: overall survival;

Supplementary Figure 3 Subgroup analysis of DFS in the combined cohort


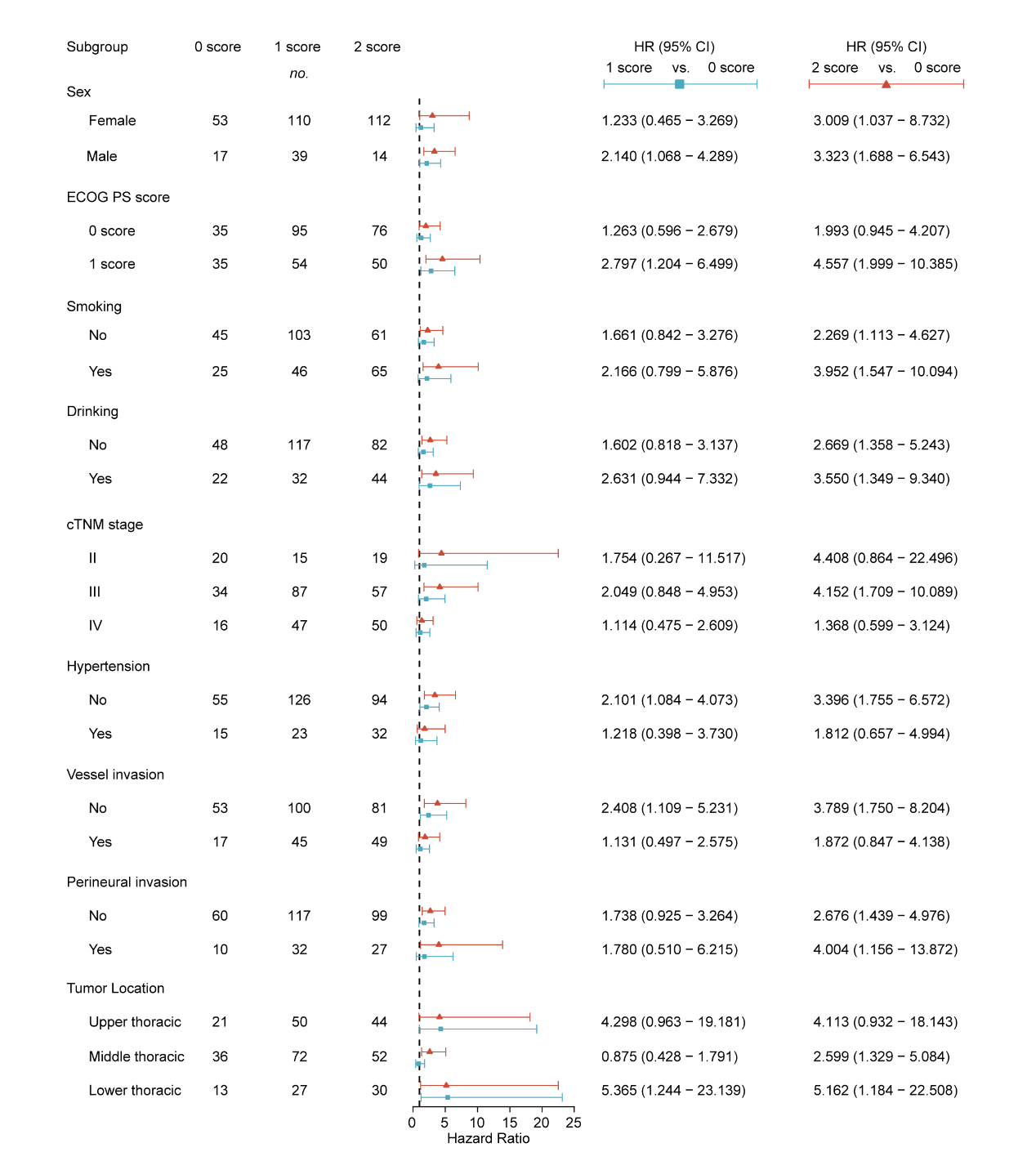


**Supplementary Figure 3** Subgroup analysis of DFS in the 0-score, 1-score, and 2-score groups in the combined cohort. Hazard ratios were derived from univariate Cox models for each subgroup. The dashed line indicates a hazard ratio of 1. 1-score group vs. 0-score group (blue), 2-score group vs. 0-score group (red). DFS, Disease-Free Survival; CI, confidence interval; HR, Hazard ratio; ECOG PS score, Eastern Cooperative Oncology Group Performance Status score; cTNM, Clinical Tumor Node Metastasis.
